# Investigating weight loss resistance across the menopausal transition: a preliminary quantitative survey of resistance-trained women

**DOI:** 10.1080/15502783.2025.2550191

**Published:** 2025-08-26

**Authors:** Anna Oelmann, John Holtje, Valeria V. Silva Ramirez, Nam Nguyen, Landon Shannahan, Gretchen Shelton, Jacey Greece, Laura Pyott, Vince Pitstick, Cassidy Bale, Bill I. Campbell

**Affiliations:** a University of South Florida, Performance & Physique Enhancement Lab, Exercise Science & Kinesiology Program, Tampa, FL, USA; b Boston University School of Public Health, Department of Community Health Sciences, Boston, MA, USA; c West Chester University, Department of Mathematics, West Chester, PA, USA; d Vidal Medical, Vidal Enterprises, St. Petersburg, FL, USA

**Keywords:** Hormones, fat loss, physiological change, menopause

## Abstract

**Background:**

Many women enter midlife unaware of the physiological and hormonal changes associated with the menopause transition. This period is commonly characterized by increased fat mass and decreased lean body mass. Women who maintain a consistent fitness lifestyle may report that previously effective fat loss strategies become less effective or potentially ineffective during this transition. This phenomenon, referred to as weight loss resistance (WLR), is defined as the inability to lose body weight or body fat despite being in a caloric deficit or engaging in activities that would normally cause weight loss, or more specifically fat loss. This survey documents the prevalence of WLR in resistance-trained women across the premenopausal, perimenopausal, and postmenopausal stages.

**Methods:**

An anonymous online survey was conducted in resistance-trained females aged 30–75 years old using Qualtrics Software (Qualtrics, Provo, UT, USA). Participants self-reported their menopause status. This preliminary analysis focused on the question: “Are you currently experiencing weight loss resistance?” with four close-ended responses: “Yes”; “No”; “I do not know”; “This does not apply to me because I am not trying to lose weight.” Only responses of “Yes” and No’ were included in the analysis. A 3 × 2 chi-square test compared WLR prevalence across the three menopausal groups. Post hoc 2 × 2 chi-square tests assessed paired-group differences.

**Results:**

About 13.7% participants identified as premenopausal, 44.5% as perimenopausal, and 41.9% as postmenopausal. The analytic sample included 1,686 participants (excluded participants included 9.3% who selected “I do not know” and 15.6% who selected “This does not apply to me because I am not trying to lose weight”). The prevalence of women who report experiencing weight loss resistance is 67.3%, 74.6%, and 80.1% for pre, peri, and post-menopausal women, respectively. Postmenopausal women self-report the highest rates of WLR compared to premenopausal (*p* < 0.001) and perimenopausal women (*p* = 0.013). Further, perimenopausal women report higher rates of WLR compared to premenopausal women (*p* = 0.034).

**Conclusion:**

These preliminary findings suggest that WLR is commonly reported among resistance-trained women across the menopause transition, increasing from premenopausal to perimenopausal and peaking in postmenopausal stages. This trend may reflect the impact of hormonal and physiological changes that hinder fat loss despite adherence to caloric deficits and increased energy expenditure. Further research is necessary to investigate the validity of these weight loss resistance claims as well as the potential disconnect between traditional weight loss strategies and midlife physiological and hormonal shifts to improve weight loss outcomes.

